# Nomogram for prediction of fatal outcome in patients with severe COVID-19: a multicenter study

**DOI:** 10.1186/s40779-021-00315-6

**Published:** 2021-03-17

**Authors:** Yun Yang, Xiao-Fei Zhu, Jian Huang, Cui Chen, Yang Zheng, Wei He, Ling-Hao Zhao, Qian Gao, Xuan-Xuan Huang, Li-Juan Fu, Yu Zhang, Yan-Qin Chang, Huo-Jun Zhang, Zhi-Jie Lu

**Affiliations:** 1 The Third Affiliated Hospital of Second Military Medical University, 225 Changhai Road, Shanghai, 200438 China; 2 The Guanggu Branch of the Women and Children’s Hospital of Hubei Province, Wuhan, 430070 China; 3 The First Affiliated Hospital of Second Military Medical University, Shanghai, 200438 China; 4 904 Hospital of PLA Joint Logistic Support Force, Wuxi, 215000 Jiangsu China; 5 Tongji Taikang Hospital, Wuhan, 430050 China; 6 924 Hospital of PLA Joint Logistic Support Force, Guilin, 541002 Guangxi China; 7 Huoshen Mountain Hospital, Wuhan, 430113 China

**Keywords:** Severe COVID-19, Nomogram, Prediction, Survival

## Abstract

**Background:**

To develop an effective model of predicting fatal outcomes in the severe coronavirus disease 2019 (COVID-19) patients.

**Methods:**

Between February 20, 2020 and April 4, 2020, consecutive confirmed 2541 COVID-19 patients from three designated hospitals were enrolled in this study. All patients received chest computed tomography (CT) and serological examinations at admission. Laboratory tests included routine blood tests, liver function, renal function, coagulation profile, C-reactive protein (CRP), procalcitonin (PCT), interleukin-6 (IL-6), and arterial blood gas. The SaO_2_ was measured using pulse oxygen saturation in room air at resting status. Independent high-risk factors associated with death were analyzed using Cox proportional hazard model. A prognostic nomogram was constructed to predict the survival of severe COVID-19 patients.

**Results:**

There were 124 severe patients in the training cohort, and there were 71 and 76 severe patients in the two independent validation cohorts, respectively. Multivariate Cox analysis indicated that age ≥ 70 years (*HR* = 1.184, 95% CI 1.061–1.321), panting (breathing rate ≥ 30/min) (*HR* = 3.300, 95% CI 2.509–6.286), lymphocyte count < 1.0 × 10^9^/L (*HR* = 2.283, 95% CI 1.779–3.267), and interleukin-6 (IL-6) >  10 pg/ml (*HR* = 3.029, 95% CI 1.567–7.116) were independent high-risk factors associated with fatal outcome. We developed the nomogram for identifying survival of severe COVID-19 patients in the training cohort (AUC = 0.900, 95% CI 0.841–0.960, sensitivity 95.5%, specificity 77.5%); in validation cohort 1 (AUC = 0.811, 95% CI 0.763–0.961, sensitivity 77.3%, specificity 73.5%); in validation cohort 2 (AUC = 0.862, 95% CI 0.698–0.924, sensitivity 92.9%, specificity 64.5%). The calibration curve for probability of death indicated a good consistence between prediction by the nomogram and the actual observation. The prognosis of severe COVID-19 patients with high levels of IL-6 receiving tocilizumab were better than that of those patients without tocilizumab both in the training and validation cohorts, but without difference (*P* = 0.105 for training cohort, *P* = 0.133 for validation cohort 1, and *P* = 0.210 for validation cohort 2).

**Conclusions:**

This nomogram could help clinicians to identify severe patients who have high risk of death, and to develop more appropriate treatment strategies to reduce the mortality of severe patients. Tocilizumab may improve the prognosis of severe COVID-19 patients with high levels of IL-6.

**Supplementary Information:**

The online version contains supplementary material available at 10.1186/s40779-021-00315-6.

## Background

Previous studies have indicated that in all coronavirus disease 2019 (COVID-19) patients, the incidence of severe cases is approximately 15% [1, 2]. The mortality rate of severe COVID-19 patients varies from 8.0 to 61.5% and significantly increases among older patients [3–8]. Early medical intervention is very important to reduce the mortality of severe patients. Thus, it is of great importance to screen out severe patients with a high risk of death promptly and accurately at the initial admission [9]. However, this is particularly difficult because of limited medical resources and staff and the large number of patients. Therefore, elucidating the independent risk factors and establishing an accurate model for predicting severe COVID-19 patients at high risk of death is necessary.

A previously established nomogram suggested five prognostic factors for predicting the outcome: Acute Physiology and Chronic Health Evaluation II (APACHE II), creatine kinase (CK), C-reactive protein (CRP), immunoglobulin A (IgA), and the interaction between CK and APACHE II [10]. However, risk factors associated with fatal outcomes in severe patients are unclear. This study aimed to provide a model to help clinicians identify patients with severe COVID-19 at high risk of death, which may be beneficial for decision making regarding treatment strategies.

## Methods

### Study population

Between February 20, 2020 and April 4, 2020, consecutive confirmed COVID-19 patients were assessed to enter into this study from three designated hospitals of COVID-19 in China: the Guanggu Branch of the Women and Children’s Hospital of Hubei Province, Tongji Taikang Hospital and Huoshen Mountain Hospital. The diagnosis of COVID-19 was based on the World Health Organization (WHO) interim guidance and guidelines for diagnosis and treatment of novel coronavirus pneumonia (5^th^ version) released by the National Health Commission of China [11, 12]. The presence of severe acute respiratory syndrome coronavirus 2 (SARS-CoV-2) in respiratory specimens was confirmed by a positive result of quantitative real-time reverse transcriptase-polymerase chain reaction (qRT-PCR) assay from nasal or pharyngeal swab specimens. Swab samples were collected and tested for SARS-CoV-2 with the Chinese Center for Disease Control and Prevention (CDC) recommended Kit (BioGerm, Shanghai, China), following WHO guidelines for qRT-PCR [13–15]. All samples were tested for SARS-CoV-2 by use of qRT-PCR with the CDC recommended Kit. The test results were confirmed by nested RT-PCR with designed primers. The nested RT-PCR assay was performed according to the previous report [16].

Severe COVID-19 group was defined if meeting at least one of the following criteria: (1) Shortness of breath, breathing rate ≥ 30/min, (2) Arterial oxygen saturation (SaO_2_, resting status) ≤ 93%, or (3) the ratio of partial pressure of arterial oxygen (PaO_2_) to fraction of inspired oxygen (FiO_2_) ≤ 300 mmHg.

During the study period, a total of 2541 patients were enrolled into this study. Of 2541 cases included in this study, 1056 patients were from the Guanggu Branch of the Women and Children’s Hospital of Hubei Province, 726 were from Tongji Taikang Hospital, and 759 were from Huoshen Mountain Hospital. According to the definition of severe COVID-19 described above, there were 124, 71, and 76 severe cases from the three hospitals, respectively. Therefore, 124 patients with severe diseases from the Guanggu Branch of the Women and Children’s Hospital of Hubei Province were included in the training cohort, while 71 and 76 severe cases in other two hospitals formed validation cohort. The selection of the study population was shown in Supplemental Fig. 1. The study was approved by the Ethics Committee of all centers. Written informed consent was waived by the Ethics Commission of each hospital for emerging infections.

### Data collection

All patients received chest computed tomography (CT) and serological examinations at admission. Laboratory tests included routine blood tests, liver function, renal function, coagulation profile, CRP, procalcitonin (PCT), interleukin-6 (IL-6), and arterial blood gas. The SaO_2_ was measured using pulse oxygen saturation in room air at resting status. Comorbidity was defined as having at least one of the followings: Hypertension, diabetes, cardiovascular disease, cerebrovascular disease, chronic lung disease, and malignant tumor for at least 6 months. All data were collected, re-checked for accuracy independently by at least two researchers.

### Statistical analysis

Continuous variables were expressed as mean (SD). Categorical variables were expressed as frequency (percentage). Categorical variables were compared by the *χ*^2^ test or Fisher’s exact test. Continuous variables were compared by the Student’s *t* test or Mann-Whitney *U* test. Survival curves were analyzed using the Kaplan-Meier method. Differences between curves were assessed using the Log-rank test.

Univariate and multivariate Cox proportional regression analysis was used for investigating the independent risk factors of death. The independent risk factors associated with the risk of mortality of patients with severe COVID-19 were used to build the nomogram in the training cohort. The performance and accuracy of the established nomogram were assessed by receiver operating characteristic (ROC) curve and calibration with 1000 bootstrap samples. The area under ROC (AUC) and optimal cut-off values were determined. Decision curve analysis (DCA) based on the net benefit was depicted by the package of *Rmda* in R. The nomogram was validated in the validation cohorts 1 and 2, respectively. The nomogram was constructed and evaluated using the R software version 3.4.1 package with the *Rms* and *Hmisc*. All statistical analysis was performed using R version 3.4.1, a *P* < 0.05 in two-tailed was the significance threshold.

## Results

### Patient clinical characteristics

Of 2541 patients included in this study, 271 patients had severe COVID-19 and 2270 were non-severe cases. Supplemental Table 1 indicated the basic characteristics of the study cohorts. Compared with non-severe cases, patients were older (*P* < 0.001) and more males (*P* < 0.001) were found in the severe disease cohort. In addition, there is more comorbidity in severe cases (*P* < 0.001). The proportion of patients with panting was higher in the severe cases (*P* < 0.001). Higher levels of white blood cell (WBC) count, neutrophil count, CRP, PCT, IL-6, total bilirubin (TBIL), alanine aminotransferase (ALT), aspartate aminotransferase (AST), lactate dehydrogenase (LDH), γ-glutamyl transpeptidase (γ-GT), and creatinine (Cr) were identified in severe cases (*P* < 0.001), while the levels of lymphocyte count, platelet (PLT), and SaO_2_ of patients with severe disease were lower than those in non-severe cases (*P* < 0.001). There were 186 patients with no manifestations of chest CT in non-severe COVID-19 cohort, and no significant differences of the findings of chest CT between severe and non-severe cases were found (*P* >  0.05). By the end of April 4, 2020, there were 58 and 39 patients died in the severe and non-severe disease group, respectively (Supplemental Table 1).

The basic characteristics of the severe patients are listed in Supplemental Table 2. The median age of the patients was 68 (range 20–100) years. One hundred and fifty-two (56.1%) patients were males and there were one hundred nineteen (43.9%) female patients. There were 73 (26.9%) patients who had a high WBC count of > 10 × 10^9^/L, and 148 (54.6%) patients with lymphopenia defined as lymphocyte count of ≤1.0 × 10^9^/L. Ninety-five (35.1%) patients had a high neutrophil count of > 6.3 × 10^9^/L, while 26 (9.6%) patients had a low PLT count of < 100 × 10^9^/L. Ground-glass opacity and consolidation were found in 122 (45.0%) and 114 (42.1%) patients, respectively. In addition, twenty-eight (10.3%) and seven (2.6%) patients had thickened interlobular septa and nodular lesions, respectively in chest CT. Of 271 severe patients, 58 died during the study period (Supplemental Table 2).

Comparison of baseline characteristics between patients in training and validation cohorts can be seen in Table 1. There were significant differences of age, proportion of smokers, incidence of panting, WBC, neutrophil count, CRP and SaO_2_ at admission between the three cohorts (*P* < 0.05). There was no significant difference in the other variables between the three cohorts (*P* >  0.05). By the end of April 4, 2020, 22 severe COVID-19 patients died in the training group, and 22 and 14 patients died in the validation group 1 and validation group 2, respectively (Table 1).

**Table 1:** Comparison of baseline characteristics between patients in training and validation cohorts [*n*(%)]

Variables	Training cohort (*n* = 124)	Validation cohort 1 (*n* = 71)	Validation cohort 2 (*n* = 76)	*P*
Age (year)				0.001
≥ 70	73 (58.9)	25 (35.2)	29 (38.2)	
< 70	51 (41.1)	46 (64.8)	47 (61.8)	
Gender				
Male	69 (55.7)	42 (59.2)	41 (54.0)	0.810
Comorbidity				0.291
Without comorbidity	31 (25.0)	27 (38.0)	26 (34.2)	
With single comorbidity	38 (30.7)	16 (22.6)	21 (27.6)	
With multiple comorbidity	55 (44.3)	28 (39.4)	29 (38.2)	
Smoke	66 (53.2)	16 (22.5)	19 (25.0)	< 0.001
Panting (breathing rate ≥ 30/min)	56 (45.2)	63 (88.7)	67 (88.2)	< 0.001
WBC (×10^9^/L)				0.005
> 10	24 (19.4)	29 (40.9)	20 (26.3)	
≤ 10	100 (80.6)	42 (59.1)	56 (73.7)	
Lymphocyte (×10^9^/L)				0.352
> 1.0	56 (45.2)	28 (39.4)	39 (51.3)	
≤ 1.0	68 (54.8)	43 (60.6)	37 (48.7)	
Neutrophil (×10^9^/L)				0.013
> 6.3	36 (29.0)	35 (49.3)	24 (31.6)	
≤ 6.3	88 (71.0)	36 (50.7)	52 (68.4)	
PLT (×10^9^/L)				0.346
≥ 100	114 (91.9)	61 (85.9)	70 (92.1)	
< 100	10 (8.1)	10 (14.1)	6 (7.9)	
CRP (mg/L)				0.026
> 10	70 (56.5)	36 (50.7)	28 (36.8)	
≤ 10	54 (43.5)	35 (49.3)	48 (63.2)	
D-dimer (mg/L)				0.992
> 0.55	81 (65.3)	46 (64.8)	50 (65.8)	
≤ 0.55	43 (34.7)	25 (35.2)	26 (34.2)	
PCT (ng/ml)				0.312
> 0.05	87 (70.2)	57 (80.3)	56 (73.7)	
≤ 0.05	37 (29.8)	14 (19.7)	20 (26.3)	
IL-6 (pg/ml)				0.298
≥ 10	68 (54.8)	47 (66.2)	44 (57.9)	
< 10	56 (45.2)	24 (33.8)	32 (42.1)	
SaO_2_ on admission				< 0.001
≥ 90%	85 (68.6)	18 (25.4)	34 (44.7)	
< 90%	39 (31.4)	53 (74.6)	42 (55.3)	
TBIL (μmol/L)				0.098
≥ 20	45 (36.3)	16 (22.5)	28 (36.8)	
< 20	79 (63.7)	55 (77.5)	48 (63.2)	
ALT (U/L)				0.363
≥ 40	35 (28.2)	22 (31.0)	16 (21.1)	
< 40	89 (71.8)	49 (69.0)	60 (78.9)	
AST (U/L)				0.633
≥ 40	41 (33.1)	19 (26.8)	25 (32.9)	
< 40	83 (66.9)	52 (73.2)	51 (67.1)	
LDH (U/L)				0.341
≥ 245	36 (29.0)	26 (36.6)	29 (38.2)	
< 245	88 (71.0)	45 (63.4)	47 (61.8)	
γ-GT (U/L)				0.262
≥ 50	64 (51.6)	28 (39.4)	36 (47.4)	
< 50	60 (48.4)	43 (60.6)	40 (52.6)	
Cr (μmol/L)				0.303
> 80	41 (33.1)	29 (40.9)	21 (27.6)	
≤ 80	83 (66.9)	42 (59.1)	55 (72.4)	
Outcomes				0.070
Dead	22 (17.7)	22 (31.0)	14 (18.4)	
Alive	102 (82.3)	49 (69.0)	62 (81.6)	

*WBC* White blood cell, *PLT* Platelet, *CRP* C-reactive protein, *PCT* Procalcitonin, *IL-6* Interleukin-6, *SaO*_*2*_ Oxygen saturation, *TBIL* Total bilirubin, *ALT* Alanine aminotransferase, *AST* Aspartate aminotransferase, *LDH* Lactate dehydrogenase, *γ-GT* γ-glutamyl transpeptadase, *Cr* Creatinine

The baseline characteristics of patients in the training cohort were shown in Table 2. There were no significant differences in gender, TBIL, ALT, AST, LDH, γ-GT, Cr, PLT, and the proportion of smokers between survivors and non-survivors (*P* >  0.05). Survivors were significantly younger than the non-survivors in the training cohort [(70.4 ± 12.3) years *vs*. (81.6 ± 7.3) years, *P* < 0.05], however, the proportion of patients with multiple comorbidities and panting (breathing rate ≥ 30/min) was significantly higher in non-survivors (*P* < 0.05). In addition, WBC and neutrophil count, CRP, D-dimer, PCT, and IL-6 were also significantly higher in non-survivors (*P* < 0.05). The lymphocyte count was significantly lower in non-survivors (*P* < 0.05).

**Table 2:** Baseline characteristics of patients in the training cohort [*n*(%)]

Variables	Death (*n* = 22)	Discharge (*n* = 102)	*P*
Gender			
Male	14 (63.6)	55 (53.9)	0.407
Comorbidity			0.005
Without comorbidity	1 (4.6)	30 (29.4)	
With single comorbidity	6 (27.3)	32 (31.4)	
With multiple comorbidity	15 (68.1)	40 (39.2)	
Smoke	13 (59.1)	53 (52.0)	0.545
Panting (breathing rate ≥ 30/min)	17 (77.3)	39 (38.2)	0.001
WBC (×10^9^/L)			0.001
> 10	10 (45.5)	14 (13.7)	
≤ 10	12 (54.5)	88 (86.3)	
Lymphocyte (×10^9^/L)			< 0.001
> 1.0	2 (9.1)	54 (52.9)	
≤ 1.0	20 (90.9)	48 (47.1)	
Neutrophil (×10^9^/L)			0.001
> 6.3	13 (59.1)	23 (22.6)	
≤ 6.3	9 (40.9)	79 (77.4)	
PLT (×10^9^/L)			0.076
≥ 100	18 (81.8)	96 (94.1)	
< 100	4 (18.2)	6 (5.9)	
CRP (mg/L)			0.008
> 10	18 (81.8)	52 (51.0)	
≤ 10	4 (18.2)	50 (49.0)	
D-dimer (mg/L)			0.006
> 0.55	20 (90.9)	61 (59.8)	
≤ 0.55	2 (9.1)	41 (40.2)	
PCT (ng/ml)			0.020
> 0.05	20 (90.9)	67 (65.7)	
≤ 0.05	2 (9.1)	35 (34.3)	
IL-6 (pg/ml)			< 0.001
≥ 10	21 (95.5)	47 (46.1)	
< 10	1 (4.5)	55 (53.9)	
SaO_2_ on admission			0.294
≥ 90%	13 (59.1)	72 (70.6)	
< 90%	9 (40.9)	30 (29.4)	
TBIL (μmol/L)			0.326
≥ 20	10 (45.5)	35 (34.3)	
< 20	12 (54.5)	67 (65.7)	
ALT (U/L)			0.147
≥ 40	9 (40.9)	26 (25.5)	
< 40	13 (59.1)	76 (74.5)	
AST (U/L)			0.213
≥ 40	10 (45.5)	31 (30.4)	
< 40	12 (54.5)	71 (69.6)	
LDH (U/L)			0.352
≥ 245	14 (63.6)	75 (73.5)	
< 245	8 (36.4)	27 (26.5)	
γ-GT (U/L)			0.441
≥ 50	13 (59.1)	51 (50.0)	
< 50	9 (40.9)	51 (50.0)	
Cr (μmol/L)			0.718
> 80	8 (36.4)	33 (32.4)	
≤ 80	14 (63.6)	69 (67.6)	

*WBC* White blood cell, *PLT* Platelet, *CRP* C-reactive protein, *PCT* Procalcitonin, *IL-6* Interleukin-6, *SaO*_*2*_ Oxygen saturation, *TBIL* Total bilirubin, *ALT* Alanine aminotransferase, *AST* Aspartate aminotransferase, *LDH* Lactate dehydrogenase, *γ-GT* γ-glutamyl transpeptadase, *Cr* Creatinine

### Independent high-risk factors associated with the fatal outcome

All variables listed in Table 1 were analyzed by univariate and multivariate Cox regression analysis. Multivariate Cox analysis indicated that age ≥ 70 years (*HR* = 1.184, 95% CI 1.061–1.321), panting (breathing rate ≥ 30/min, *HR* = 3.300, 95% CI 2.509–6.286), lymphocyte count < 1.0 × 10^9^/L (*HR* = 2.283, 95% CI 1.779–3.267), and IL-6 >  10 pg/ml (*HR* = 3.029, 95% CI 1.567–7.116) were independent risk factors associated with fatal outcomes (Table 3).

**Table 3:** Univariate and multivariate COX proportional hazards regression analysis of death in the training cohort

Variable	Univariate		Multivariate	
	*HR* (95% CI)	*P*	*HR* (95% CI)	*P*
Age (≥70 years *vs*. < 70 years)	9.245 (2.054–11.624)	0.004	1.184 (1.061–1.321)	0.003
Gender (male *vs*. female)	1.495 (0.577–3.874)	0.407	–	–
Comorbidity			–	**–**
Without comorbidity	1		1	
With single comorbidity	3.625 (0.639–4.503)	0.120	1.810 (0.794–2.585)	0.551
With multiple comorbidity	2.250 (1.407–4.947)	0.022	1.155 (0.831–3.130)	0.479
Smoke (yes *vs*. no)	1.309 (0.533–3.212)	0.556	–	–
Panting (breathing rate ≥ 30/min) (yes *vs*. no)	5.492 (2.876–7.078)	0.002	3.300 (2.509–6.286)	0.004
WBC (> 10 × 10^9^/L *vs*. ≤ 10 × 10^9^/L)	5.238 (1.906–9.397)	0.001	2.046 (0.726–4.503)	0.524
Lymphocyte (< 1.0 × 10^9^/L *vs*. ≥ 1.0 × 10^9^/L)	5.263 (2.513–9.615)	0.002	2.283 (1.779–3.267)	0.011
Neutrophil (> 6.3 × 10^9^/L *vs*. ≤ 6.3 × 10^9^/L)	4.961 (1.884–8.068)	0.001	2.439 (0.717–3.768)	0.392
PLT (≥ 100 × 10^9^/L *vs*. < 100 × 10^9^/L)	3.556 (0.911–6.876)	0.068	–	–
CRP (> 10 mg/L *vs*. ≤ 10 mg/L)	4.327 (1.369–7.677)	0.013	1.214 (0.721–2.211)	0.196
D-dimer (> 0.55 mg/L *vs*. ≤ 0.55 mg/L)	6.721 (1.490–9.319)	0.013	1.395 (0.668–3.268)	0.195
PCT (> 0.05 ng/ml *vs*. ≤ 0.05 ng/ml)	3.224 (1.154–6.645)	0.032	2.255 (0.768–4.767)	0.118
IL-6 (> 10 pg/ml *vs*. ≤ 10 pg/ml)	4.547 (3.184–8.659)	0.002	3.029 (1.567–7.116)	0.009
SaO_2_ on admission(≥ 90% *vs*. < 90%)	1.662 (0.739–2.007)	0.180	–	**–**
TBIL (≥ 20 μmol/L *vs*. < 20 μmol/L)	1.595 (0.627–2.057)	0.327	–	–
ALT (≥ 40 U/L *vs*. < 40 U/L)	2.024 (0.775–5.283)	0.150	–	–
AST (≥ 40 U/L *vs*. < 40 U/L)	1.909 (0.746–4.883)	0.177	–	–
LDH (≥ 245 U/L *vs*. < 245 U/L)	1.923 (0.739–5.007)	0.180	–	–
γ-GT (≥ 50 U/L *vs*. < 50 U/L)	1.444 (0.567–3.677)	0.440	–	**–**
Cr (> 80 μmol/L *vs*. ≤ 80 μmol/L)	1.195 (0.456–3.129)	0.717	–	–

*WBC* White blood cell, *PLT* Platelet, *CRP* C-reactive protein, *PCT* Procalcitonin, *IL-6* Interleukin-6, *SaO*_*2*_ Oxygen saturation, *TBIL* Total bilirubin, *ALT* Alanine aminotransferase, *AST* Aspartate aminotransferase, *LDH* Lactate dehydrogenase, *γ-GT* γ-glutamyl transpeptadase, *Cr* Creatinine, *HR* Hazard ratio, *95% CI* 95% confidence interval

### Survival analysis in the patients with the high level of IL-6

Due to the high level of IL-6 correlating with poor outcomes in severe COVID-19 patients, the therapeutic effect of tocilizumab in the patients with high IL-6 was further analyzed. In the training cohort, it was demonstrated that the prognosis of patients receiving tocilizumab was better than that of patients not receiving tocilizumab, but without significance (*P* = 0.105, Supplemental Fig. 2a). Similar results were also observed in the validation cohort 1 and validation cohort 2, respectively (*P* = 0.133, *P* = 0.210, Supplemental Fig. 2b-c).

### Construction and validation of the nomogram

**Fig. 1:**
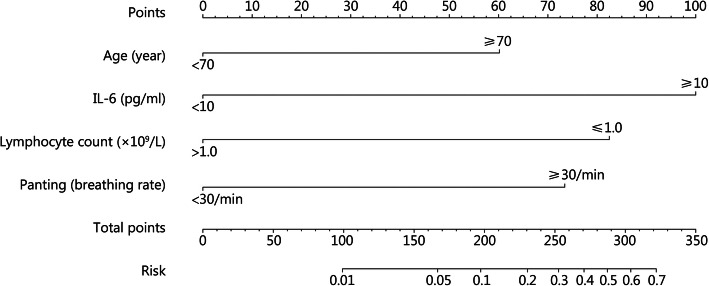
Risk prediction nomogram for patients with COVID-19

**Fig. 2:**
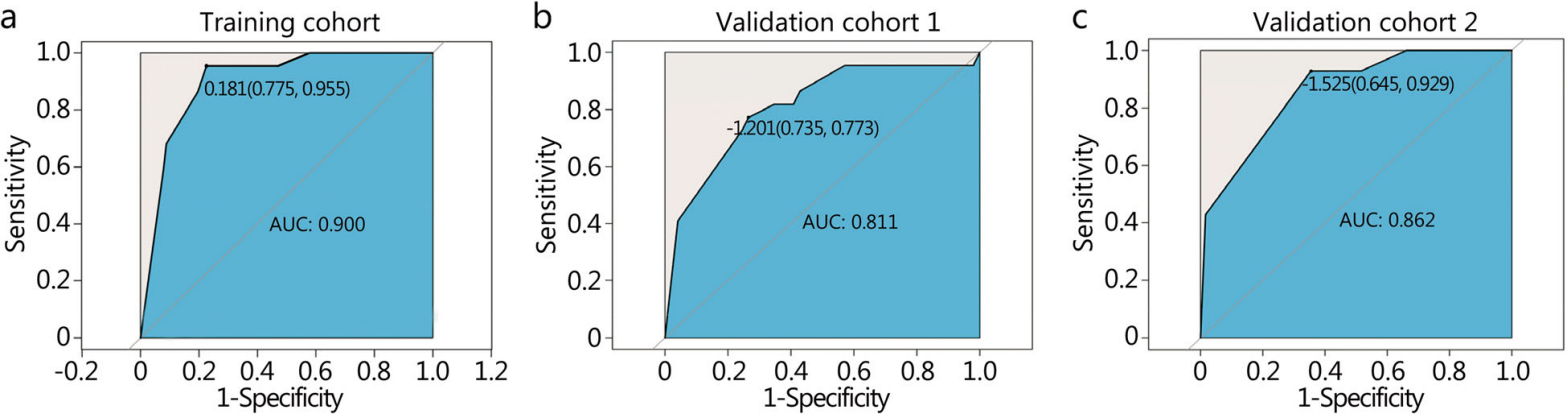
The receiver operating characteristic (ROC) curves of the nomogram in the training cohort **a**, validation cohort 1 **b**, and validation cohort 2 **c**

**Fig. 3:**
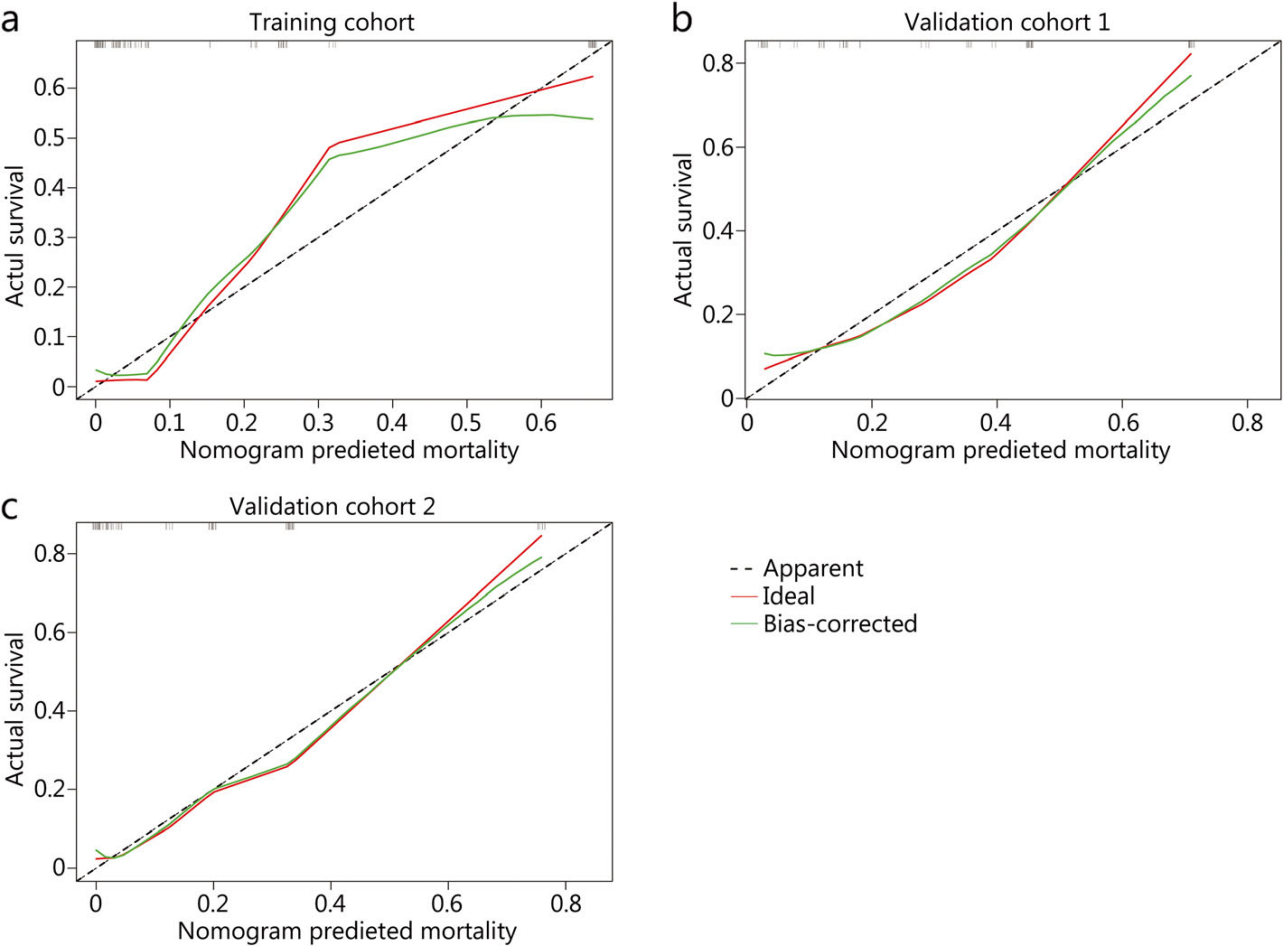
The calibration curves of the nomogram in the training cohort **a**, validation cohort 1 **b**, and validation cohort 2 **c**

Four independent risk factors found to be associated with the risk of mortality of patients in the multivariate analyses were incorporated into the nomogram (Fig. 1). The ROC curve was employed to assess the predictive ability of the established nomogram, and the result demonstrated that the AUC was 0.900 (95% CI 0.841–0.960) in the training cohort, with a sensitivity of 95.5% and specificity of 77.5% (Fig. 2a). Moreover, the calibration curves for nomogram predicted mortality indicated that a good consistency between observed actual outcomes and predicted ones in the training cohort (Fig. 3a).

In the validation cohort 1, the AUC was 0.811 (95% CI 0.763–0.961) for patients with a sensitivity of 77.3% and specificity of 73.5% (Fig. 2b). In the validation cohort 2, the AUC was 0.862 (95% CI 0.698–0.924) for patients with a sensitivity of 92.9% and specificity of 64.5% (Fig. 2c). The calibration curves also showed good agreement between prediction and observation in the risk of mortality in the two validation cohorts (Fig. 3b-c).

### Clinical application of the nomogram

**Fig. 4:**
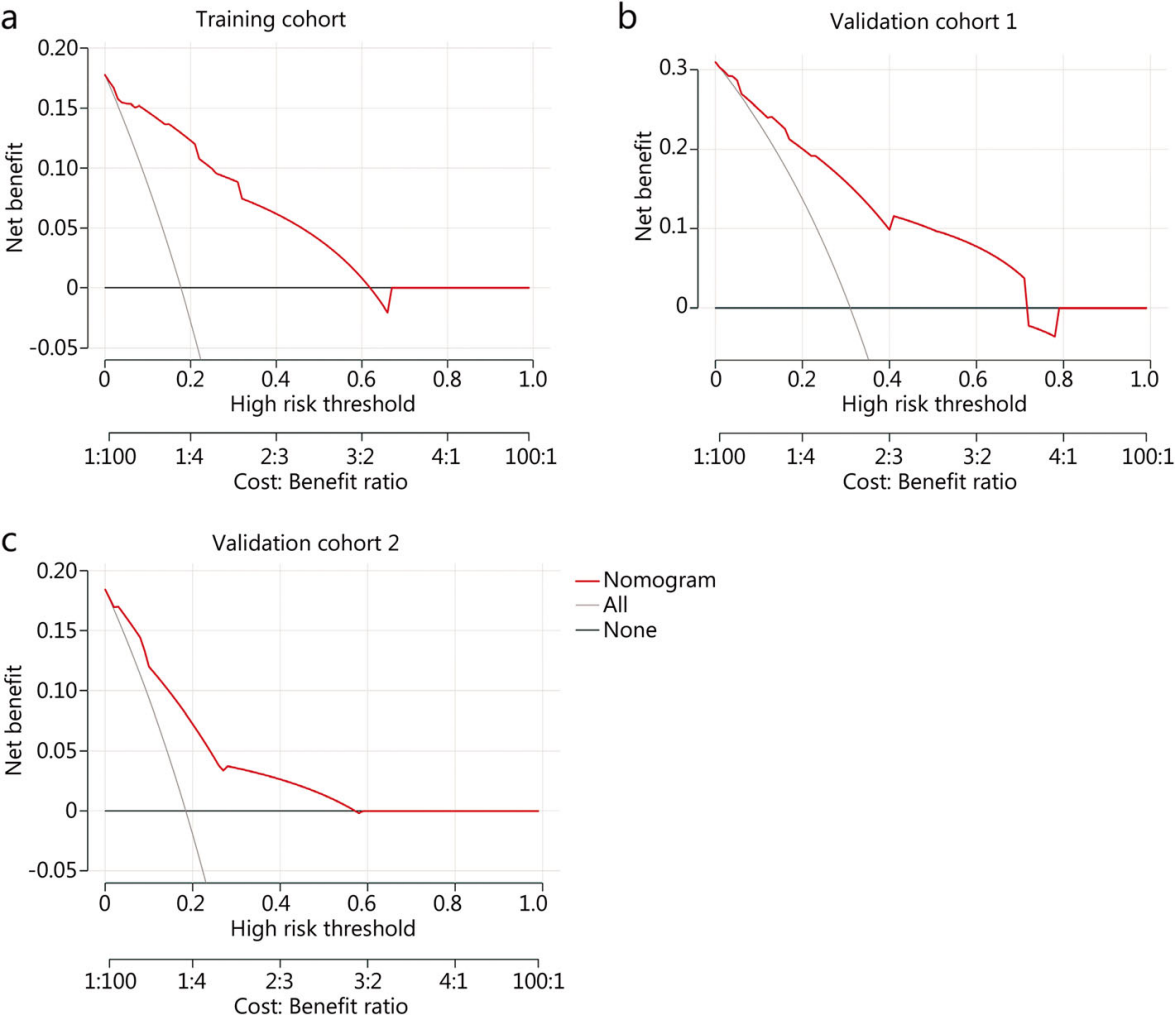
Decision curve analysis of the nomogram of patients with COVID-19. DCA compares the net benefits of three scenarios in predicting the risk of mortality: A perfect prediction model (grey line), screen none (horizontal solid black line), and screen based on the nomogram (ride line). The DCA curves were depicted in the training cohort **a**, validation cohort 1 **b**, and validation cohort 2 **c**.

DCA based on the net benefit and threshold probabilities was performed to assess the clinical applicability of the risk prediction nomogram. The DCA showed that our risk prediction nomogram had a superior net benefit with a wide range of threshold probabilities in the training cohort and validation cohorts (Fig. 4).

## Discussion

Our study revealed the clinical characteristics and risk factors for fatal outcomes in confirmed severe COVID-19 patients based on multicenter cohorts. Multivariate Cox analysis in this study indicated that age, lymphopenia, respiratory rate ≥ 30/min, and IL-6 were independent high-risk factors associated with poor prognosis. Older age has been proven to be a risk factor for the virus infection and survival in many previous studies [17–20]. Elderly patients with severe COVID-19 were more likely to develop fatal outcomes because of the rapid progression of the disease, which reminded us of providing early intervention for elderly severe patients. Similarly, lymphopenia was more common in the nonsurvivors and severe cases according to previous reports, suggesting dysregulation of the immune response in patients with COVID-19 [21–23]. Nevertheless, most of these were only descriptive studies. A study clarified that lower lymphocyte count was predictive of COVID-19 progression [24], whereas the impact of lymphocyte count on the survival of severe COVID-19 was unclear. This study demonstrated that a lymphocyte count < 1.0 × 10^9^/L was independently associated with death in severe cases.

Recent studies have found that the cytokine storm is an important factor leading to rapid disease progression and poor prognosis [25, 26]. IL-6 is one of the major cytokines involved in cytokine storms [27, 28]. A previous univariate analysis showed that the IL-6 level was associated with worse survival without significance [18]. Our study showed that a high level of IL-6 was a predictor of death in severe COVID-19 patients. Additionally, the survival curve showed that the outcome was better in patients with tocilizumab than in patients without tocilizumab in the training cohort. Nonetheless, no significant difference was found. Similar results were also found in validation cohort 1 and validation cohort 2. The reason for this result may be attributable to the small sample size. Although the previous study has shown that tocilizumab can reduce mortality, high-quality studies are still needed to verify the effectiveness of tocilizumab on the survival of patients with severe COVID-19 [29].

Increasing respiratory rate is an important clinical feature of acute respiratory distress syndrome (ARDS), which is a major cause of death in severe COVID-19 patients [8, 17, 30]. A previous study showed that a respiratory rate ≥ 24/min was a risk factor for death in the univariate analysis [18], whereas no significance was found after the multivariate regression analysis. Our multivariate regression analysis clarified that a respiratory rate ≥ 30/min was a predictor of death. For patients with increasing respiratory rates, especially those with respiratory rate ≥ 30/min, it is necessary for physicians to be aware of the potential progression of ARDS.

Many previous studies have shown that comorbidity was significantly associated with high mortality rate and disease progression [24, 31]. Nevertheless, in this study, the significance of comorbidity was only indicated in the univariate analysis, but not in the multivariate regression analysis, which may be ascribed to different patients enrolled in these studies. All the patients included in this study had severe COVID-19, and the proportion of comorbidities was approximately 70%, which was significantly higher than those in other studies.

Previous studies have shown several prediction models with different parameters [1, 24, 31, 32]. Compared with other studies, the predictive effect of age, panting, and lymphopenia has been described in previous reports, while the main feature of this study is the analysis of the prognostic value of IL-6 in severe COVID-19 patients for the first time. IL-6 plays an important role in the pathophysiological changes of severe cases. However, the prediction value of IL-6 was not shown in other prediction models. In addition, our study also indicated that tocilizumab, a monoclonal antibody against the IL-6 receptor, may improve the prognosis of severe COVID-19 patients with the high level of IL-6.

Our study has some limitations. First, this is a retrospective study, and there may be potential biases in the selection of patients. Second, the sample size of the study was relatively small, and the results need to be further validated in a larger cohort.

## Conclusion

This study firstly developed a nomogram for predicting fatal outcomes in the severe COVID-19 patients. The four predictors included in the model are easy to obtain. The prediction risk of the model indicated a good consistency with the observed one. Hence, this nomogram may be conducive to more effective treatment to reduce the mortality of those severe cases at high risk of death.

## Supplementary Information

**Additional file 1: Supplemental Figure 1** Selection of the study population.

**Additional file 2: Supplemental Figure 2** Survival curves of severe COVID-19 patients with the high level of IL-6 receiving tocilizumab and not receiving tocilizumab treatment. **a** The survival curve of severe COVID-19 patients with the high level of IL-6 receiving tocilizumab and not receiving tocilizumab treatment in the training cohort (*P* = 0.105). **b** The survival curve of severe COVID-19 patients with the high level of IL-6 receiving tocilizumab and not receiving tocilizumab treatment in the validation cohort 1 (*P* = 0.133). **c** The survival curve of severe COVID-19 patients with the high level of IL-6 receiving tocilizumab and not receiving tocilizumab treatment in the validation cohort 2 (*P* = 0.210).

**Additional file 3: Supplemental Table 1** Comparison of Baseline characteristics between severe patients and non-severe patients *n*(%). WBC. White blood cell; PLT. Platelet; CRP. C-reactive protein; PCT. Procalcitonin; IL-6. Interleukin-6; SaO_2_. Oxygen saturation; TBIL. Total bilirubin; ALT. Alanine aminotransferase; AST. Aspartate aminotransferase; LDH. Lactate dehydrogenase; γ-GT. γ-glutamyl transpeptadase; Cr. Creatinine; CT. Computed tomography. **Supplemental Table 2** Baseline characteristics of the patients [*n*(%)]. ^a^Ages are shown as median (range). WBC. White blood cell; PLT. Platelet; CRP. C-reactive protein; PCT. Procalcitonin; IL-6. Interleukin-6; SaO_2_. Oxygen saturation; TBIL. Total bilirubin; ALT. Alanine aminotransferase; AST. Aspartate aminotransferase; LDH. Lactate dehydrogenase; γ-GT. γ-glutamyl transpeptadase; Cr. Creatinine; CT. Computed tomography

## Data Availability

The patient level data has been uploaded to the Figshare database. Dataset. 10.6084/m9.figshare.12866060.v1.

## Abbreviations

COVID-19: Coronavirus disease 2019
SARS-CoV-2: Severe acute respiratory syndrome coronavirus 2
APACHE II: Acute Physiology and Chronic Health Evaluation II
CK: Creatine kinase
CRP: C-reactive protein
IgA: Immunoglobulin A
qRT-PCR: Quantitative real-time reverse transcriptase-polymerase chain reaction
CT: Computed tomography
WBC: White blood cell
PLT: Platelet
PCT: Procalcitonin
IL-6: Interleukin-6
SaO_2_: Oxygen saturation
TBIL: Total bilirubin
ALT: Alanine aminotransferase
AST: Aspartate aminotransferase
LDH: Lactate dehydrogenase
γ-GT: γ-glutamyl transpeptadase
Cr: Creatinine
*HR*: Hazard ratio
95% CI: 95% Confidence interval
ARDS: Acute respiratory distress syndrome.
