# Supplementary figures and images for: Nomogram for prediction of fatal outcome in patients with severe COVID-19: a multicenter study
Source: Mil Med Res. 2021 Mar 17;8:21. doi: 10.1186/s40779-021-00315-6 (PMC7967101; doi:10.1186/s40779-021-00315-6)

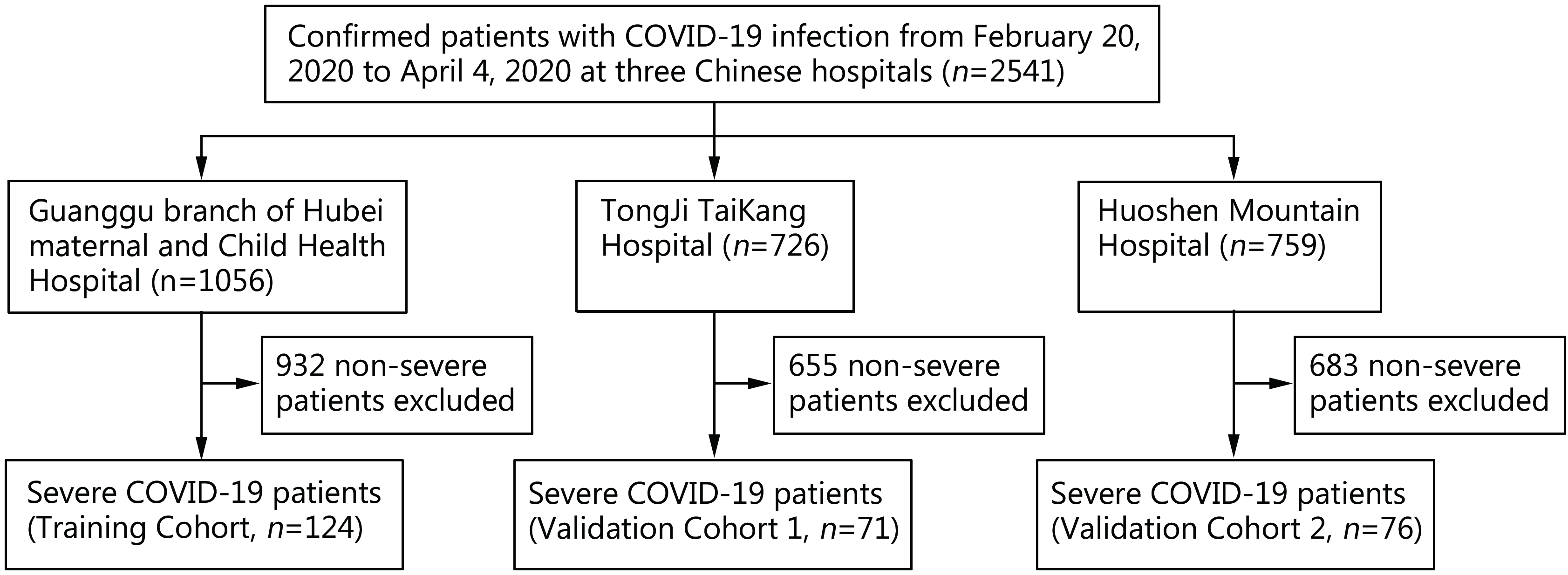

Supplement: Supplementary file 1 — Additional file 1: Supplemental Figure 1 Selection of the study population. [file 40779_2021_315_MOESM1_ESM.tif]

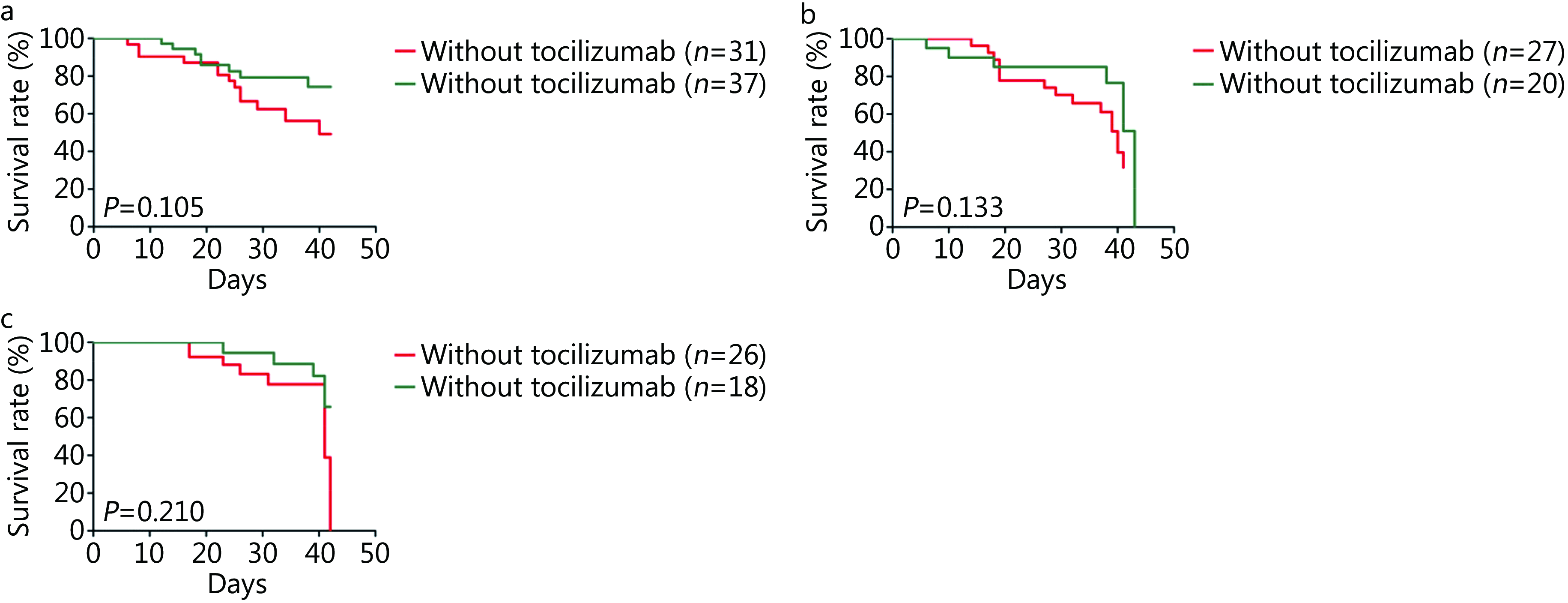

Supplement: Supplementary file 2 — Additional file 2: Supplemental Figure 2 Survival curves of severe COVID-19 patients with the high level of IL-6 receiving tocilizumab and not receiving tocilizumab treatment. a The survival curve of severe COVID-19 patients with the high level of IL-6 receiving tocilizumab and not receiving tocilizumab treatment in the training cohort (P = 0.105). b The survival curve of severe COVID-19 patients with the high level of IL-6 receiving tocilizumab and not receiving tocilizumab treatment in the validation cohort 1 (P = 0.133). c The survival curve of severe COVID-19 patients with the high level of IL-6 receiving tocilizumab and not receiving tocilizumab treatment in the validation cohort 2 (P = 0.210). [file 40779_2021_315_MOESM2_ESM.tif]
