# Supplementary material for: Nomogram for prediction of fatal outcome in patients with severe COVID-19: a multicenter study
Source: Mil Med Res. 2021 Mar 17;8:21. doi: 10.1186/s40779-021-00315-6 (PMC7967101; doi:10.1186/s40779-021-00315-6)
Supplement: Supplementary file 3 — Additional file 3: Supplemental Table 1 Comparison of Baseline characteristics between severe patients and non-severe patients n(%). WBC. White blood cell; PLT. Platelet; CRP. C-reactive protein; PCT. Procalcitonin; IL-6. Interleukin-6; SaO2. Oxygen saturation; TBIL. Total bilirubin; ALT. Alanine aminotransferase; AST. Aspartate aminotransferase; LDH. Lactate dehydrogenase; γ-GT. γ-glutamyl transpeptadase; Cr. Creatinine; CT. Computed tomography. Supplemental Table 2 Baseline characteristics of the patients [n(%)]. aAges are shown as median (range). WBC. White blood cell; PLT. Platelet; CRP. C-reactive protein; PCT. Procalcitonin; IL-6. Interleukin-6; SaO2. Oxygen saturation; TBIL. Total bilirubin; ALT. Alanine aminotransferase; AST. Aspartate aminotransferase; LDH. Lactate dehydrogenase; γ-GT. γ-glutamyl transpeptadase; Cr. Creatinine; CT. Computed tomography [file 40779_2021_315_MOESM3_ESM.docx]

**Supplemental Table 1**  Comparison of Baseline characteristics between severe patients and non-severe patients *n*(%)

| Variables | Severe patients (*n*=271) | Non-severe patients (*n*=2270) | *P* |
| --- | --- | --- | --- |
| Age (year) |  |  | <0.001 |
| ≥70 | 127(46.9) | 435(19.2) |  |
| <70 | 144(53.1) | 1835(80.8) |  |
| Gender |  |  |  |
| Male | 152(56.1) | 925(40.8) | <0.001 |
| Female | 119(43.9) | 1345(59.2) |  |
| Comorbidity |  |  | <0.001 |
| Without comorbidity | 84(31.0) | 1354(59.7) |  |
| With single comorbidity | 75(27.7) | 615(27.1) |  |
| With multiple comorbidity | 112(41.3) | 301(13.2) |  |
| Smoke |  |  | 0.030 |
| Yes | 101(37.3) | 1003(44.2) |  |
| No | 170(62.6) | 1267(55.8) |  |
| Panting (breathing rate ≥ 30/min) |  |  | <0.001 |
| Yes | 186(68.6) | 380(16.7) |  |
| No | 85(31.4) | 1890(83.3) |  |
| WBC (×10^9^/L) |  |  | <0.001 |
| >10 | 73(26.9) | 61(2.7) |  |
| ≤10 | 198(73.1) | 2209(97.3) |  |
| Lymphocyte (×10^9^/L) |  |  | <0.001 |
| >1.0 | 123(45.4) | 2037(89.7) |  |
| ≤1.0 | 148(54.6) | 233(10.3) |  |
| Neutrophil (×10^9^/L) |  |  | <0.001 |
| >6.3 | 95(35.1) | 117(5.2) |  |
| ≤6.3 | 176(64.9) | 2153(94.8) |  |
| PLT (×10^9^/L) |  |  | <0.001 |
| ≥100 | 245(90.4) | 2228(98.2) |  |
| <100 | 26(9.6) | 42(1.8) |  |
| CRP (mg/L) |  |  | <0.001 |
| >10 | 134(49.5) | 230(10.1) |  |
| ≤10 | 137(50.5) | 2040(89.9) |  |
| D-dimer (mg/L) |  |  | 0.489 |
| >0.55 | 177(65.3) | 1434(63.2) |  |
| ≤0.55 | 94(34.7) | 836(36.8) |  |
| PCT (ng/ml) |  |  | <0.001 |
| >0.05 | 200(73.8) | 462(20.4) |  |
| ≤0.05 | 71(26.2) | 1808(79.6) |  |
| IL-6 (pg/ml) |  |  | <0.001 |
| ≥10 | 159(58.7) | 143(6.3) |  |
| <10 | 112(41.3) | 2127(93.7) |  |
| SaO_2_ on admission |  |  | <0.001 |
| ≥90% | 137(50.6) | 2270(100.0) |  |
| <90% | 134(49.4) | 0(0.0) |  |
| TBIL (μmol/L) |  |  | <0.001 |
| ≥20 | 89(32.8) | 211(9.3) |  |
| <20 | 182(67.2) | 2059(90.7) |  |
| ALT (U/L) |  |  | <0.001 |
| ≥40 | 73(26.9) | 197(8.7) |  |
| <40 | 198(73.1) | 2073(91.3) |  |
| AST (U/L) |  |  | <0.001 |
| ≥40 | 85(31.4) | 105(4.6) |  |
| <40 | 186(68.6) | 2165(95.4) |  |
| LDH (U/L) |  |  | <0.001 |
| ≥245 | 91(33.6) | 97(4.3) |  |
| <245 | 180(66.4) | 2173(95.7) |  |
| γ-GT (U/L) |  |  | <0.001 |
| ≥50 | 128(47.2) | 69(3.0) |  |
| <50 | 143(52.8) | 2201(97.0) |  |
| Cr (μmol/L) |  |  | <0.001 |
| >80 | 91(33.6) | 8(0.4) |  |
| ≤80 | 180(66.4) | 2262(99.6) |  |
| Findings of chest CT |  |  | 0.067 |
| Ground-glass opacity | 122(45.0) | 977(43.1) |  |
| Consolidation | 114(42.1) | 872(38.4) |  |
| Thickened interlobular septa | 28(10.3) | 114(5.0) |  |
| Nodular lesions | 7(2.6) | 121(5.3) |  |
| No findings of chest CT | 0 | 186(8.2) |  |
| Outcome |  |  | <0.001 |
| Dead | 58(21.4) | 39(1.7) |  |
| Alive | 213(78.6) | 2231(98.3) |  |

WBC. White blood cell; PLT. Platelet; CRP. C-reactive protein; PCT. Procalcitonin; IL-6. Interleukin-6; SaO_2_. Oxygen saturation; TBIL. Total bilirubin; ALT. Alanine aminotransferase; AST. Aspartate aminotransferase; LDH. Lactate dehydrogenase; γ-GT. γ-glutamyl transpeptadase; Cr. Creatinine; CT. Computed tomography

**Supplemental Table 2**  Baseline characteristics of the patients [*n*(%)]

| Variables | Total patients (*n*=271) |
| --- | --- |
| Age (year)^a^ | 68(20-100) |
| Gender |  |
| Male | 152(56.1) |
| Female | 119(43.9) |
| Comorbidity |  |
| Without comorbidity | 84(31.0) |
| With single comorbidity | 75(27.7) |
| With multiple comorbidity | 112(41.3) |
| Smoke |  |
| Yes | 101(37.3) |
| No | 170(62.7) |
| Panting (breathing rate ≥ 30/min) |  |
| Yes | 186(68.6) |
| No | 85(31.4) |
| WBC (×10^9^/L) |  |
| >10 | 73(26.9) |
| ≤10 | 198(73.1) |
| Lymphocyte (×10^9^/L) |  |
| >1.0 | 123(45.4) |
| ≤1.0 | 148(54.6) |
| Neutrophil (×10^9^/L) |  |
| >6.3 | 95(35.1) |
| ≤6.3 | 176(64.9) |
| PLT (×10^9^/L) |  |
| ≥100 | 245(90.4) |
| <100 | 26(9.6) |
| CRP (mg/L) |  |
| >10 | 134(49.5) |
| ≤10 | 137(50.5) |
| D-dimer (mg/L) |  |
| >0.55 | 177(65.3) |
| ≤0.55 | 94(34.7) |
| PCT (ng/ml) |  |
| >0.05 | 200(73.8) |
| ≤0.05 | 71(26.2) |
| IL-6 (pg/ml) |  |
| ≥10 | 159(58.7) |
| <10 | 112(41.3) |
| SaO_2_ on admission |  |
| ≥90% | 137(50.6) |
| <90% | 134(49.4) |
| TBIL (μmol/L) |  |
| ≥20 | 89(32.8) |
| <20 | 182(67.2) |
| ALT (U/L) |  |
| ≥40 | 73(26.9) |
| <40 | 198(73.1) |
| AST (U/L) |  |
| ≥40 | 85(31.4) |
| <40 | 186(68.6) |
| LDH (U/L) |  |
| ≥245 | 91(33.6) |
| <245 | 180(66.4) |
| γ-GT (U/L) |  |
| ≥50 | 128(47.2) |
| <50 | 143(52.8) |
| Cr (μmol/L) |  |
| >80 | 91(33.6) |
| ≤80 | 180(66.4) |
| Findings of chest CT |  |
| Ground-glass opacity | 122(45.0) |
| Consolidation | 114(42.1) |
| Thickened interlobular septa | 28(10.3) |
| Nodular lesions | 7(2.6) |
| Outcome |  |
| Dead | 58(21.4) |
| Alive | 213(78.6) |

^a^Ages are shown as median (range). WBC. White blood cell; PLT. Platelet; CRP. C-reactive protein; PCT. Procalcitonin; IL-6. Interleukin-6; SaO_2_. Oxygen saturation; TBIL. Total bilirubin; ALT. Alanine aminotransferase; AST. Aspartate aminotransferase; LDH. Lactate dehydrogenase; γ-GT. γ-glutamyl transpeptadase; Cr. Creatinine; CT. Computed tomography
